# Exploring the impact of clonal definition on B-cell diversity: implications for the analysis of immune repertoires

**DOI:** 10.3389/fimmu.2023.1123968

**Published:** 2023-04-17

**Authors:** Aurelien Pelissier, Siyuan Luo, Maria Stratigopoulou, Jeroen E. J. Guikema, María Rodríguez Martínez

**Affiliations:** ^1^ IBM Research Europe, Rüschlikon, Switzerland; ^2^ Department of Biosystems Science and Engineering, ETH Zurich, Basel, Switzerland; ^3^ Department of Pathology, Amsterdam University Medical Centers, location AMC, Lymphoma and Myeloma Center Amsterdam (LYMMCARE), Amsterdam, Netherlands

**Keywords:** B-cell, repertoire, clone, diversity, clustering, analysis, RNA, antibody

## Abstract

The adaptive immune system has the extraordinary ability to produce a broad range of immunoglobulins that can bind a wide variety of antigens. During adaptive immune responses, activated B cells duplicate and undergo somatic hypermutation in their B-cell receptor (BCR) genes, resulting in clonal families of diversified B cells that can be related back to a common ancestor. Advances in high-throughput sequencing technologies have enabled the high-throughput characterization of B-cell repertoires, however, the accurate identification of clonally related BCR sequences remains a major challenge. In this study, we compare three different clone identification methods on both simulated and experimental data, and investigate their impact on the characterization of B-cell diversity. We observe that different methods lead to different clonal definitions, which affects the quantification of clonal diversity in repertoire data. Our analyses show that direct comparisons between clonal clusterings and clonal diversity of different repertoires should be avoided if different clone identification methods were used to define the clones. Despite this variability, the diversity indices inferred from the repertoires’ clonal characterization across samples show similar patterns of variation regardless of the clonal identification method used. We find the Shannon entropy to be the most robust in terms of the variability of diversity rank across samples. Our analysis also suggests that the traditional germline gene alignment-based method for clonal identification remains the most accurate when the complete information about the sequence is known, but that alignment-free methods may be preferred for shorter sequencing read lengths. We make our implementation freely available as a Python library cdiversity.

## 1 Introduction

Antibodies are protective proteins produced by B cells in response to the presence of foreign pathogens, they have an exceptional ability to recognize a wide variety of target antigens and can display exquisite binding specificity (1). To ensure broad antigen recognition, antibodies undergo several rounds of maturation where selected B cells gain increased affinity, avidity, and anti-pathogen activity, while the rest are eliminated through apoptosis.

Briefly, B cell receptors (BCRs) are assembled through the rearrangement of the V, D, and J gene segments, coupled with stochastic insertions and deletions of nucleotides at the gene boundaries, i.e. at the V and D, and the D and J gene *junctions* (2). The junctions between the V, D, and J gene segments are known as the complementary determining region 3 (CDR3). This region is the most diverse part of the BCR sequence and plays a crucial role in determining the binding specificity to foreign antigens (3).

Once a BCR has been formed, B cells are exposed to antigens in the secondary lymphoid organs and undergo affinity maturation in microanatomical structures known as Germinal Centers (GCs) (4). Through antigen-driven competition, selected B cells receive secondary signals that direct them to undergo further rounds of cellular replication and BCR diversification through somatic hypermutation (SHM). Through this process, B cells with higher affinity to the target antigen are preferentially selected to further multiply, while the ones with lower affinity undergo programmed apoptosis. This process results in the progressive expansion and evolution of the initial pool of founder cells into distinct groups of clonally related B cells (referred to as B-cell clones) that compete against each other for antigen-mediated survival signals. Through an accelerated Darwinian process of diversification and selection, some of these clones expand significantly and can become dominant, while others disappear (5).

Because of the stochastic nature underlying clonal selection coupled with the randomness associated with experimental BCR sampling and sequencing, it is common to observe a fraction of B cells without any clonally related B-cell in a repertoire. In this manuscript, we refer to this group of B cells as *singletons*, and use the term *non-singletons* to refer to B cells that have other clonally related B cells. Of course, the distinction between *singletons* and *non-singletons* depends not only on the random experimental cell sampling and repertoire sequencing depth, but also depends crucially on the user-defined threshold to define and separate clonally related and unrelated cells, as will be discussed in Section 2.2.

The rapid change and adaptation of B-cell repertoires in response to antigen stimulation driven by internal and external immune insults makes the sequencing and analysis of BCRs a valuable tool to characterize the immune status of an individual. Furthermore, as memory B cells produced during short-lived immune episodes can survive for a very long time, sometimes for the entire lifetime of a person, their analysis can also reveal information about the past and current pathogens encountered by an individual (6, 7). Beyond infections, the analysis of B-cell repertoires can provide valuable fingerprints of an individual’s immunological status, and enable the diagnosis of complex diseases, chronic inflammatory conditions, allergies, responses to vaccination, etc (8–15).

Advances in Adaptive Immune Receptor Repertoire Sequencing (AIRR-Seq) technologies have considerably increased the amount of repertoire data that is available for analysis and improved our understanding of the dynamics of B-cell repertoires in both individuals and populations. Typical B-cell repertoire analyses start by grouping BCR sequences into clones of related B cells. The reconstruction of phylogenetic lineages of clonally related B cells provides information about the evolutionary paths that led to the development of functional antibodies and it is also useful to understand the progression of diseases such as chronic infections, autoimmune diseases or cancer. In most cases, however, immune repertoire data show significant differences in clonal composition across individuals in humans and mice (16), and even, between identical twins (17). This variability makes the direct comparison of sequence repertoires across individuals inadequate to identify robust immune repertoire-based signatures.

A more promising approach to comparing immune repertoires across individuals focuses on the investigation of sequence-independent quantifiers such as clonal diversity indices. These quantifiers offer the possibility of correlating immune repertoire diversity to immunological status and, in doing so, readily allow for immune-repertoire-based comparisons across individuals. Nevertheless, there is still substantial ambiguity and a lack of quantitative understanding of the effectiveness of the diversity metrics to reliably capture status-specific information from immune repertoires. Realistic measures of diversity should reflect not only the relative abundances of clones, but also the main differences between them (18, 19). Furthermore, the use of specific diversity indices, such as the Shannon (20) or Simpson (21) diversity indices may yield qualitatively different results in different contexts (18, 19). Intuitively, that is because these indices do not put the same weights on the clone abundances in the repertoire. For example, the richness score is most sensitive to the rarest clones, while the Simpson index (probability that two randomly selected individuals belong to different species) and the dominance score are affected mainly by the most common clones.

The limitations associated with individual metrics have supported the practice of aggregating multiple indices for immunological classification. An example of such aggregation is the Hill-based diversity profile, which integrates a continuum of single diversity indices and can facilitate a global quantification of the immunological information contained in immune repertoires (22, 23). Nevertheless, the estimation of these indices and profiles is heavily influenced by the sequencing depth of the experiment. For example, species richness quantifies the number of species in a sample, and not surprisingly, shows strong correlations with the number of BCR sequences available in the repertoire. Several bias estimators have been proposed to correct for incomplete sample information such as the Chaos estimator (24, 25). However, these estimators present vulnerabilities that limit their applicability to immune repertoires with variable sequencing depth (26). For instance, the Chaos estimator relies heavily on the correct quantification of singletons and doubletons, which is often prone to error in the context of B-cell repertoires.

In fact, another challenge in the analysis of B-cell repertoire data is the grouping of BCR into clones. Theoretically, a group of clonally related B cells represents a group of B cells descending from the same common ancestor (aka founder cell). In an experimental context, the founder cell has long disappeared, being replaced by better-adapted descendants. Therefore, in most cases reconstructing phylogenetic trees amounts to inferring trees where the founder cell is unknown. In practice, B cells with similar characteristics, such as the same gene segments and similar CDR3, are grouped together in the same clone. This *empirical definition* of clone poses several challenges, the first being the arbitrariness of the choice of BCR properties used to define a clone, and second, the subjective choice of metric and threshold used to separate clonally related and unrelated B cells. Importantly, the optimal way to group clones may differ depending on the dataset.

In this context, a variety of methods has been proposed to (semi-)automatically identify clones from a set of BCR sequences. Some are based on probabilistic models that infer a hypothetical unmutated common ancestor to be used as tree root, which enables the inference of rooted trees interpreted as clones (27, 28). The most common techniques rely on CDR3 sequence similarities as well as the alignment of BCRs to reference V and J gene germline sequences (29–33). As these alignments are prone to error, some recent approaches leverage natural language processing (NLP) techniques to define similarity indicators independent of these gene alignments (34).

**Our contribution**: Each method for clonal identification depends on arbitrary choices of BCR sequence features, sequence-based distances and thresholds, and therefore, may lead to substantially different clonal groupings – besides displaying high variability in computational complexity and robustness. This variability might affect the estimation of clonal diversity which, as we discussed earlier, is crucial for the global analysis of B-cell repertoire data. In this work, we consider different definitions of clones and investigate the consistency and robustness of commonly used diversity indices at different sequencing depths and across different samples and technical replicates. By objectively comparing the performance and consistency of both clone definitions and diversity indices in various experimental contexts, we aim to investigate how the different empirical clone definitions affect diversity analyses and the biological conclusions extracted from them. Finally, to facilitate the use of the different clonal identification methods and diversity metrics, we make our implementation freely available as a python library (Section 4.4.3).

## 2 Results

### 2.1 B cell repertoire data

To characterize the influence of the different metric and clone definition choices on repertoire diversity analyses, we collected three different B-cell repertoire datasets from three different biological contexts:

- **Simulated data.** We collected artificial repertoires with known clonal relationships (34), generated by randomly adding mutations based on learned lineage tree topologies from a multiple sclerosis (MS) study (35). Thus, the repertoires exhibit wide variability in terms of sequence diversity, junction lengths, and clone sizes. While the artificial generation may bias the repertoire towards certain patterns used for its generation, they provide essential information about both the B cells clonal relationships and their lineage history. From this ground truth information, we can compute exact diversity indices, which is fundamental to quantitatively assess the accuracy of each clonal definition method and metric. This information is inaccessible in experimental datasets.
- **Germinal center data.** The second dataset is a collection of B-cell repertoires from 10 individual GCs extracted from the same lymph node of a patient with chronic sialadenitis (36). Two replicates per GCs are available. Importantly, the first 70 nucleotides of the V gene segments are missing due to the experimental design. Primers were designed to bind within the FR1 region, and were thus removed during the read processing to avoid PCR or sequencing errors (37). This limitation makes this dataset particularly interesting for our study, as it enables us to assess the impact of an uncertain V gene assignment on diversity estimations. Furthermore, as GCs can be considered semi-independent evolutionary structures with limited cell exchanges, they exhibit high variability in B-cell diversities even within the same lymph node (29). Therefore, the comparisons of technical replicates from the same GC allow us to establish confidence values for our inferred diversity estimators, as samples extracted from the same GC are expected to exhibit a similar degree of diversity compared to samples from other GCs.
- **Vaccination data.** Our third dataset comes from a study of hepatitis B-associated chronic infection and vaccination responses (8). This dataset characterizes the different B-cell repertoire landscapes of individuals shortly after vaccination and/or infection compared to controls (non-vaccinated and non-infected individuals). The dataset contains 27 samples from controls, infected individuals, as well as pre- and post- (2 weeks) vaccinated individuals.

**Figure 1:**
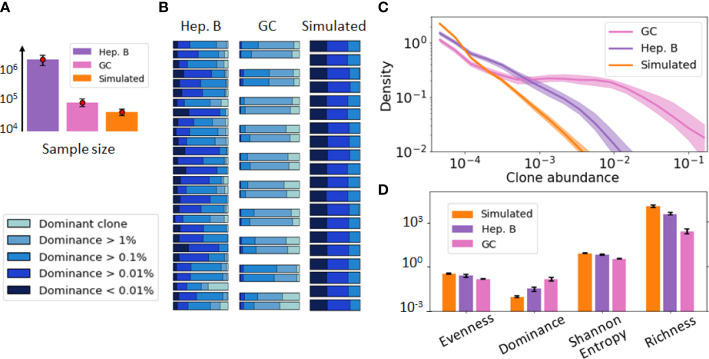
Visualization of the B-cell repertoires dataset used in our study, including a germinal center (GC) [36], hepatitis B (Heb.B) [5] and simulated [27] datasets. In this figure, clones have been identified according to previous conventions [50, 19] (shared V, J gene segments and similar CDR3s). **(A)** Average sample size of the three datasets, where the error bar represents one standard deviation. **(B)** Stacked histogram depicting the proportion of sequences belonging to the dominant clone, expanded clones (dominance > 1% excluding the dominant one), and non-expanded clones (dominance < 1%) in each sample of each dataset. Replicate samples in the GC repertoire dataset are grouped together. **(C)** Clonal abundance distribution in the three repertoires after normalizing the 3 datasets to account for sample differences. The solid line represents the distribution averaged over all samples and the shaded area indicates one standard deviation. **(D)** Computed diversity indices for each dataset of the previous figure. The error bar represents one standard deviation.

**Figure 1** provides a visualization of these three datasets. For this initial data exploration, we followed previous conventions (29, 30). Namely, we grouped B-cells sequences into clones if they share the same V and J gene segments as well as exhibit more than 90% CDR3 sequence similarity. As these datasets involve repertoires of different sizes (**Figure 1A**), we performed the comparative analysis after subsampling all the repertoires to the same size (30k sequences) to remove any potential bias due to sample size in the comparison. A dominance stacked histogram (**Figure 1B**) shows that the 3 datasets display very different clonal compositions. This is more evident in the abundance density plot (**Figure 1C**), where the GC data exhibits a *plateau* of clones with similar abundance, while still including highly dominant clones ( >10% ). Contrary to this pattern, the simulated data does not have any dominant clone, and the most abundant clone reaches only 0.5% abundance. These differences are corroborated by commonly used diversity indices such as richness (38), Shannon entropy (20) or evenness tephill1973diversity (**Figure 1D**), thus highlighting the relevance of these metrics to inform about the clonal composition of these datasets.

### 2.2 Different clonal identification methods yield inconsistent B-cell groups

The first step in the analysis of B-cell repertoire data is the grouping (or clustering) of BCRs into clonal families. In this work, we focus on three clonal identification approaches previously described in the literature.

- **Junction-based methods:** In this method, B cells are assigned to the same clone if and only if their receptors share the same CDR3 sequence. This has the advantage of being computationally simple and eliminating any ambiguity when setting arbitrary clustering thresholds. On the other hand, since the junctions of clonally related B cells typically exhibit small sequence differences due to SHM, this approach tends to split branches of the same lineage into different clones, leading to an inflation of the diversity metrics. Sequencing errors further contribute towards artificially increasing the number of clones.
- **Alignment-based methods:** A more commonly used approach relies on the junction sequence *and* the V and J assignments (39). We refer to this method as VJ & Junction. Namely, B cells are assigned to the same clone if their receptors share the same V and J gene segments and their CDR3 sequence similarity is above a predefined threshold. CDR3 similarity is typically assessed with the Levenshtein distance (40) and the threshold is set around 90%, with some variability depending on the dataset. Hence, this method allows for small sequence divergences in clonally related cells due to potential insertions and deletions through the subsequent rounds of B-cell diversification through SHM and sequencing errors.

**Figure 2:**
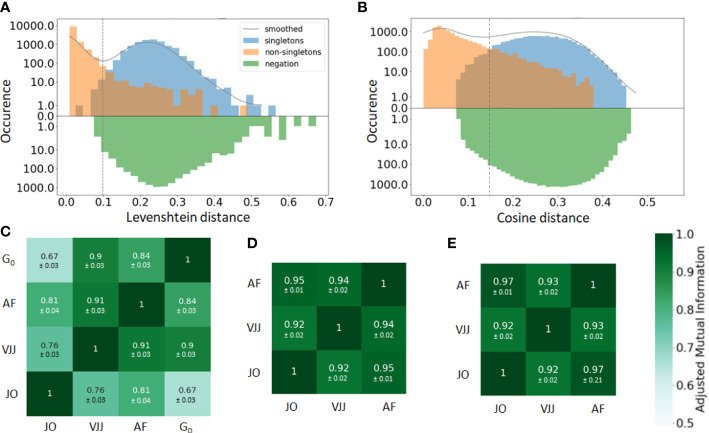
**(A, B)** Distance to the nearest neighbor sequence distribution, both within the same repertoire (blue and orange) and to the negation sequences (green). The distances to nearest neighbors are labeled according to the ground truth (singleton or non-singletons), i.e. the clonal groups in the simulated data. Results are shown for both the **(A)** alignment-based method, i.e. based on common V-J segments and junction similarity, and **(B)** alignment-free method, based on the cosine distance between *k*-mers frequency vectors of each BCR (in the plot, *k*=7). The choice between alignment-based and alignment-free methods results in clearly different distributions of pairwise distances. The distance threshold used to define separate clusters is displayed for each clonal identification method (dashed vertical line). **(C–E)** Adjusted mutual information comparing the clustering partitions obtained from the three methods: *Junction-only* (JO), *VJ & Junction* (VJJ), *Alignment-free* (AF) and *Ground-truth* ( $G_{0}$ ). Results are averaged over all samples and provided for the **(C)** simulated, **(D)** germinal center, and **(E)** hepatitis B dataset. Each cell shows the average adjusted mutual information and the standard deviation. The ground truth of clonal assignment is only available for the simulated dataset (Subplot C).

In practice, the similarity threshold is adjusted for each dataset independently. There are several ways of doing so. A first, intuitive approach consists in computing the distances between pairs of junctions from B cells with the same V and J gene segments (**Figure 2A**). The distribution of pairwise distances is expected to be the mixture of two distributions, one corresponding to distances between members of the same clone (non-singleton sequences) and the second corresponding to distances between clonally unrelated sequences (singletons). The value that separates the two modes of the distribution can then be used as a threshold to separate both clonally-related and unrelated sequences (41) (**Figure 2A**).

The bi-modality-based threshold has however a high computational cost. An alternative method assumes that clones do not span multiple individuals. Hence, sequences randomly sampled from multiple unrelated individuals, i.e. *negation sequences*, can be introduced and used to define a threshold by computing the distribution of distances between negation sequences and their closest counterparts within the considered individual (42) (**Figure 2A**). In practice, a threshold is chosen that allows a fraction of false-positive sequences roughly equal to a tolerance δ to be below the chosen threshold. This heuristic aims for high specificity, which is approximately 1−δ . In this work, the threshold was set using a tolerance of δ=1% . a tolerance set to 1%.

Finally, the threshold and the computed CDR3 pairwise Levenshtein distances are used together with the Hierarchical Agglomerative Clustering (HAC) algorithm (43) to further split BCRs with the same V and J gene segments into different clonal groups (39).

1. **Alignment free methods:** As germline gene alignments are error-prone, alignment-based methods might fail to identify clonal relatives accurately, especially when part of the nucleotides are missing in the sequences (as in the GC dataset described in section 2.3). To overcome this limitation, an alignment-free method that leverages NLP techniques has been recently introduced (34). In brief, the method decomposes each BCRs into k -mers (substrings of length k ) and uses the term frequency-inverse document frequency(*tf-idf*) as a weighting scheme that increases proportionally to the number of times a k -mer term appears in the document but is offset by the frequency of the term in the corpus. The logic behind this is to emphasize rare and hopefully meaningful terms while reducing the influence of common and uninformative terms.

Once a vectorized representation of each BCR has been built, BCR similarities are computed with the cosine distance, which allows for a very fast computation of similarities among text strings. The pairwise distances are then fed into the HAC algorithm to compute the final clusters, with a distance threshold defined from the negation sequences in the same way as with the *alignment-based* method (**Figure 2B**).

A straightforward way to visualize the consistency and performance of the different clonal identification methods is through the classification of singletons. As mentioned earlier, singletons are clonally unrelated sequences, and thus, we expect them to exhibit larger distances to their nearest repertoire neighbors than non-singletons sequences. We first run a comparative analysis in the simulated dataset, as we have ground truth information about clonal relationships. **Figures 2A, B** displays the distance to the nearest neighbors of each B cell in the simulated data, for both singletons (blue) and non-singletons sequences (orange). We observe that both methods fail to identify accurately some of the singletons in the simulated dataset (8% for alignment-free, and 1% for VJ & Junction). These inaccuracies can be visualized with the (i) blue sequences to the left of the vertical line and (ii) orange sequences to the right of the vertical line (note the log-scale on the y -axis). Overall, our analysis suggests that the VJ & Junction method (alignment based) performs the best at classifying singletons in the simulated data.

While we do not know the true clonal assignment of the experimental datasets, we observe that all methods disagree within their respective classification of singletons (SI section 1). To better quantify the similarity of the inferred clonal group across methods, we computed the adjusted mutual information (AMI) (44) between the clusters inferred with each method and for each dataset (**Figures 2C–E**). AMI is a variation of mutual information that compares the partitions produced by different clustering schemes. Furthermore, AMI also corrects for the effect of the agreement between clusters solely due to chance. A value close to 0 indicates no overlap, while a value of 1 corresponds to identical cluster partitions. As expected from the differences in clonal definitions used by the three methods, we observe differences in the clusters inferred by each method. Focusing first on the synthetic dataset for which the ground truth is known (**Figure 2C**), the VJ & Junction method performs best and achieves an AMI with the ground truth of 0.9, while the junction-only method has the lowest performance with an AMI of 0.67. This is an illustration of how a small sequence dissimilarity tolerance in the CDR3 is beneficial to faithfully reconstruct clonal families. Interestingly, the AMI between the three methods is higher in the GC and Hepatitis B dataset (AMI >0.92 ). That is likely because in these datasets, there are a few abundant clones with many identical junctions, thus inflating slightly the AMI between the three methods (dominance ∼10% *vs* ∼1% for the simulated dataset).

Importantly, additional analysis revealed that these differences are not due to chance or subsampling, as the three methods were found to be giving similar clonal relationships after subsampling even across different sample sizes and shuffling (**Figure S2** in SI section 2). In fact, because of the HAC algorithm and *tf-idf* k -mer representations used for the clonal identification, the same two sequences may or may not be in the same cluster depending on the rest of repertoire sequences. This supports our hypothesis that diversity quantification significantly depends on the method used for clonal identification.

### 2.3 V and J gene segments may be misaligned, impacting the clonal identification accuracy in alignment-based methods

**Figure 3:**
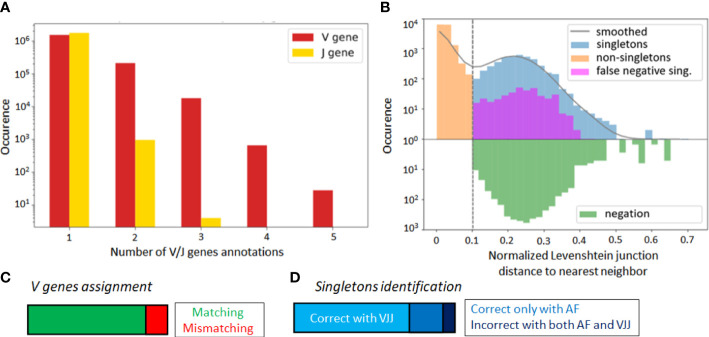
**(A)** Occurrence rate of B-cell sequence with multiple V and J gene segment annotations of equivalent alignment score after the IgBlast query in the GC dataset. **(B)** Distance to nearest distribution for B-cell sequences in the alignment-based (VJ& Junction method) clonal identification method in the GC dataset. False negative singletons originating from V or J gene misalignment are depicted in purple. **(C)** Proportion of matched and mismatched V gene assignment between the original and truncated simulated dataset (where the first 70 nucleotides were artificially removed). **(D)** Correctly and wrongly assigned singletons by the VJJ and AF method on the simulated dataset.

Alignment-based methods for clonal identification rely on the correct calling of the germline V and J gene segments to the BCR sequences. Unfortunately, V gene assignments can be ambigious, especially for shorter read lengths (45). More concretely, in the GC dataset, the first 70 nucleotides of V gene segments are missing due to the experimental design. Therefore, it is possible that a non-negligible portion of V genes is incorrectly called, and this could bias the clonal characterization of this dataset when using the VJ & Junction (alignment-based) method. Such limitations, originating from the use of FR1-binding primers (37), lead to sequencing errors in that region that are common in next-generation sequencing experiments (36, 37). To further investigate this hypothesis, we looked for sequences with ambiguous V and J gene annotations in the IgBlast output (46), whereby ambiguity was defined as having multiple gene matches with equivalent alignment scores. We found that 13% of assigned V genes were ambiguous, as compared to only 0.05% of J genes (**Figure 3A**).

To further test for the impact of these potential V gene misalignments, we considered the singleton sequences of the GC dataset. For each singleton identified in our repertoire, we looked at the top 6 V and J gene annotations, and checked how often one of these annotations reassigned the sequence to an existing cluster. We refer to these singletons as *potential false negatives*, because the alignment-based method has labeled them as singletons, while there is significant likelihood that they belong to an existing clone. Strikingly, we found that 8.8% of singletons inferred by the VJ & Junction method in the GC dataset were potential false negative (depicted in purple on **Figure 3B**, note the log-scale in the y axis). Interestingly, the alignment-free correctly classified 83% of the 8.8% identified false negatives (depicted in purple in **Figure S3**). This suggests that, while the VJ & Junction method results in better clustering assignments when the full sequence information is available, the alignment-free method might be preferable for shorter sequences and especially, when the germline V gene alignment is ambiguous.

To further investigate this, we artificially removed the 70 first nucleotides of the V region in each sequence of the simulated dataset. In this way, we replicated the experimental sequencing limitations of the GC dataset, while still having ground truth labels to accurately identify false negatives. Averaging across samples, 16% of the sequences were assigned to an incorrect V gene (**Figure 3C**), which led to a significant decrease in the average sample AMI between the VJJ method and the ground truth (from 0.90±0.3 to 0.79±0.05 , while the AF method resulted in an AMI of 0.84±0.03 ), thus confirming the negative impact of wrong V gene assignment on clone identification. Regarding singletons (**Figure 3D**), we observed that 27% of the singletons identified by the VJ & Junction method on the truncated data were actually false negatives (while this rate is less than 1% with the correct gene assignments). Among these false negatives, the AF method correctly assigned 72% as non-singletons, thus also supporting the use of alignment-free methods with ambiguous V gene calls.

### 2.4 Sensitivity of diversity indices to clonal identification methods

In the previous section, we showed how inferring clonal relationships in B-cell repertoires markedly depends on the method used. We now investigate how these variabilities affect repertoire comparisons when characterized by different diversity indices. We calculated sample diversity using various diversity metrics for all samples and all three clonal identification methods. As diversity metrics, we considered dominance (47), richness (38), Simpson index (21), Shannon entropy (20), Hill’s diversity (22) profiles. We also used Chao statistical estimators for richness and Shannon entropy (24, 25, 48) which account for incomplete sample information (see Methods Section 4.4.3).

**Figure 4:**
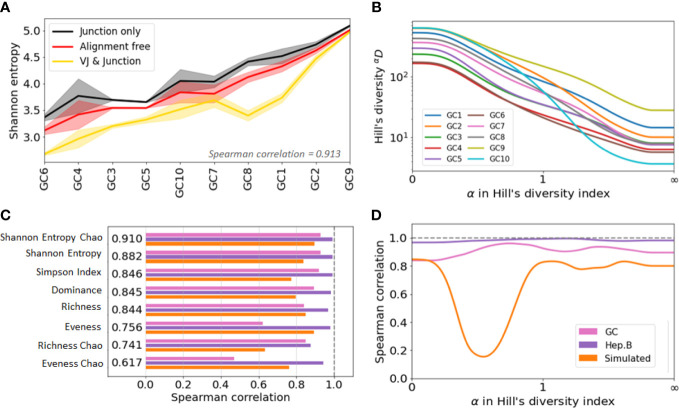
Agreement analysis of diversity indices across different clone identification methods. **(A)** Shannon entropy diversity index for each GC, listed from least to most diverse. As there are two replicates for each GC, the solid line represent the mean value between the two replicates, and the shaded area highlights the min and max values. **(B)** Spearman correlation between the diversity indices obtained with the three clonal identification methods. The computed correlation is shown for the three datasets analyzed in our study. The figure shows the average correlation across the three datasets. **(C)** Hill’s Diversity profiles of each GC (averaged over the two replicated) with clones obtained from the alignment free method (the x-axis has been transformed with an exponential tangent function for visual clarity). **(D)** Mean Spearman correlation between the diversity indices obtained with the three clonal identification methods.

Our analyses showed that the three clone identification methods result in differences in the diversity indices larger than one standard deviation (**Figure 4A**, SI section 5). Metrics such as dominance or Simpson index, which put less weight on rare species, were less affected by the clonal identification method than those sensitive to rare clones such as richness. This is logical, as these metrics pay less attention to low frequency clones that might be the result of incorrect sequence assignment to larger clones. In general, however, one should exert caution when comparing diversity indices of B-cell clone repertoires if different diversity indices were used.

Interestingly, although the indices differ in value, they show similar patterns of variation across samples and across clonal identification methods. **Figure S3A** shows the Shannon entropy profiles across different samples when clones are computed with the three different clonal identification methods, and **Figure 4A** shows the Shannon entropy with replicates grouped together to quantify the variability across both samples and replicates. These figures clearly illustrate that, although the Shannon entropy values are numerically different depending on the method used to identify the clones, the rank of entropy values across samples follows similar trends. In the case of the GC dataset, the variability across GCs seems higher than the variability across replicates (**Figure 4A**). This implies that some underlying patterns of the repertoire clonal structure are captured by all clone identification methods, and are thus reflected in the diversity indices. We quantified this trend by computing the Spearman correlation coefficient of the diversity indices. A Spearman value close to 1 indicates that the two tested methods lead to highly similar diversity-based sample ranks. To quantify rank similarity in different biological contexts, we computed the correlation across methods and across GC replicates for all diversity indices (**Supplementary Table S1**). We perform similar cross-method analyses on the other two datasets and provide all the computed correlation values in the **Supplementary Table S1**.

Interestingly, the correlation scores vary drastically depending on the dataset and evaluation method. These differences are partly explained by the variability of the diversity indices across samples, where the mean/std ranging ranges from 1 to 80 (SI section 5). Intuitively, it is more difficult to rank confidently the samples when their diversity index values are closer to each other. Still, we found that the Chao estimator for Shannon entropy yields the best performance with a Spearman correlation ≥0.8 for all tested comparisons. This is depicted **Figure 4B**, where we averaged the Spearman correlation across each pair of clonal identification methods. It shows that the Chao estimator for Shannon entropy yields the highest averaged correlation over the three datasets ( ρ=0.910 ). Other metrics also reveal high levels of correlation with the exception of the evenness, which only exhibits a correlation of ∼ 0.7 (0.756 and 0.617 for the Chao estimator). Evenness being the ratio of two quantities, it is more sensitive to variability in the richness and entropy estimation. Also, the Chao estimator for richness showed lower correlation than the richness itself. As the Chao correction formula relies heavily on the number of identified singletons, a potential cause behind this low performance is the unreliable detection of singletons during clonal identification.

Rather than a single diversity index, the B-cell repertoire landscape may also be characterized in terms of diversity profiles (23) (**Figures 4C**, **S4**). Under Hill’s unified diversity framework (22), the diversity index of order α is defined as:


(1) $${\;}^{\alpha }\text{D}={\left(\sum_{i=1}^{S}p_{i}^{\alpha }\;\right)}^{1/\left(1-\alpha \right)}$$

where $p_{i}$ is the relative abundance of species i and $\sum^{}p_{i}=1$ . Values of α<1 tend to favor rare species, while values of α>1 favor the most common species. The advantage of the Hill’s unified diversity index is that it provides a unified representation of the most common diversity indices, which can be recovered for different values of α , including richness ( ${\;}^{0}\text{D}$ ), dominance ( $1{/}^{\infty }\text{D}$ ), Shannon entropy ( $\log\;{[}^{1}\text{D}]$ ) and the Simpson index ( $1{/}^{2}\text{D}$ ).

We computed the Spearman correlation of the α diversity indices across the different clonal identification methods and investigated how the choice of α affects the correlation of the diversity indices by setting values of α between 0 and 100 with steps of 0.01. We also investigated whether there is an optimal α that leads to a maximum value of correlation (**Figure 4D**). Interestingly, the optimal α parameter is different for the three datasets studied: $\alpha_{\text{opt}}=0.58$ for GC data, $\alpha_{\text{opt}}=0.85$ for Hepatitis B, and $\alpha_{\text{opt}}=1.47$ for the simulated data. Overall, we found that the value of α that maximizes the Spearman correlation averaged over the three datasets to be $\alpha_{\text{opt}}=0.97$ . This is in good agreement with **Figure 4B**, which indicates that among all the diversity metrics tested, the Shannon entropy and its Chao corrected variant are the optimal indicators. As a reminder, the Shannon entropy ( H ) is closely related to the Hill’s diversity of parameter α=1 ( $H=\log\;{[}^{1}\text{D}]$ ), while other computed indicators are related to ${\;}^{0}\text{D}$ , ${\;}^{2}\text{D}$ and ${\;}^{y}\text{D}$ .

Finally, we observe a substantial drop in the correlation in **Figure 4D** for the simulated data. Computing the diversity profile of the simulated repertoires revealed that Hill’s diversity values are near equal across all samples ( ±1% ) for values of α∈[0.1,0.9] (**Figure S3B**). This low variability, possibly coming from an unrealistic simulated environment (not enough variability for the low abundance clones), could potentially explain why the correlation is lower for these values of α .

### 2.5 Sensitivity of clonal identification and diversity metrics to sequencing depth

Another interesting question is the influence of sequencing depth (i.e. sample size) in clonal identification and diversity characterization. It is also worth investigating how effective traditional statistical estimators are, as the Chao estimators for richness and Shannon entropy to minimize the bias associated with sample size variability. To investigate these aspects, we sub-sampled repertoire sequences with sampling ratios from 1% to 100%, and evaluated the changes in clonal identification performance for different subsampling fractions on the simulated data. Interestingly, the clustering performances were not affected by the subsampling, with the AMI between the inferred clusters and the ground truth staying roughly constant for subsampling fractions higher than 2% (SI section 6).

**Figure 5:**
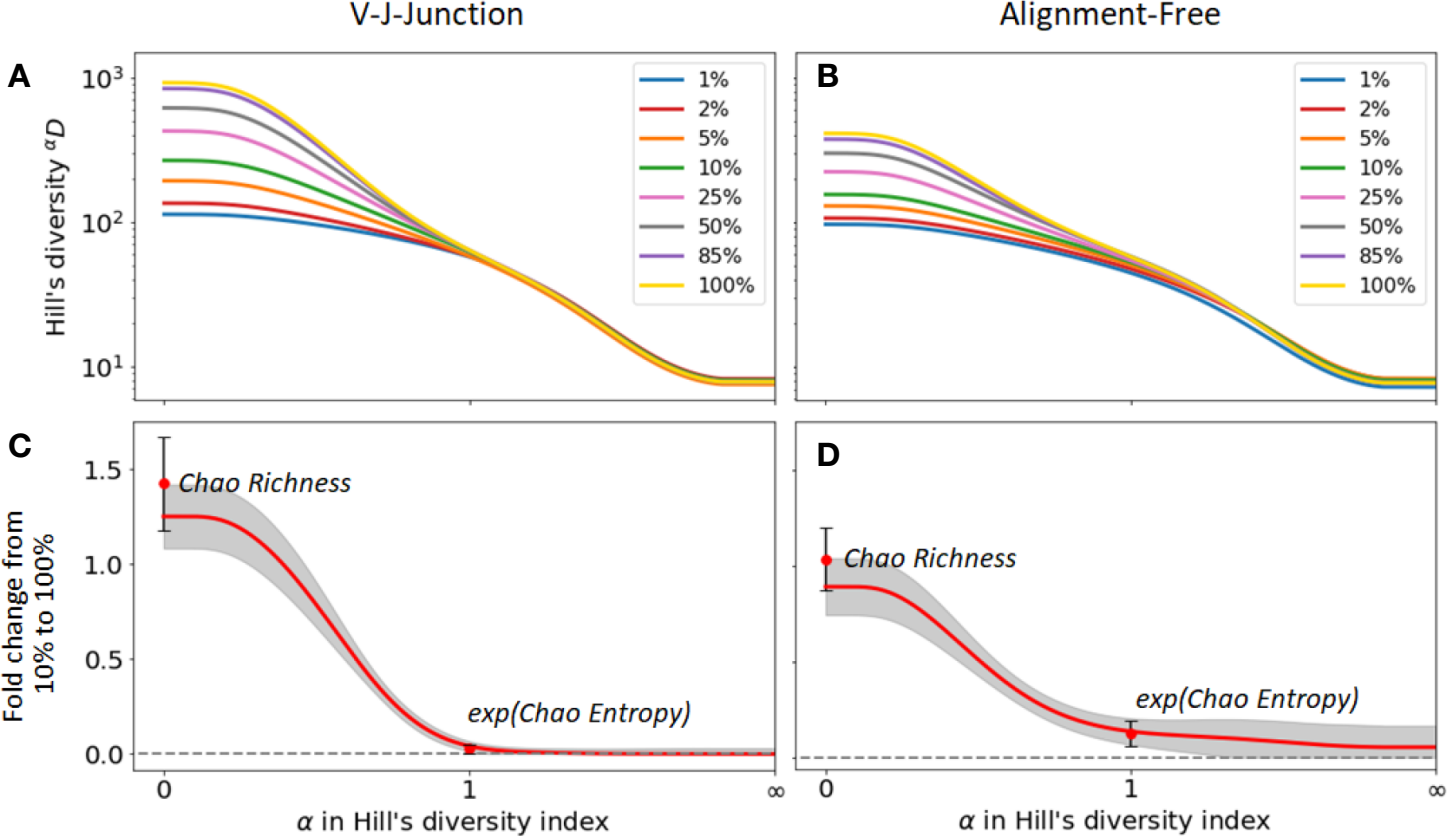
Sensitivity of diversity metrics to sequencing depth for the GC dataset. **(A, B)** Hill’s diversity profile calculated at different sub-sampling ratios varying from 1% to 100%. **(C, D)** Fold changes between Hill’s diversity index computed respectively at 10% sub-sampling and 100% sampling. The mean (read line) and one standard deviation (grey shaded area) across the 100 sub-sampling repetitions are shown in both figures. Similarly, the mean and standard deviation of the Chao estimator for richness and entropy are shown.

Next, we evaluated the change in diversity indices for different samples sizes when different clonal identification methods are used. For that, we computed the fold change between the diversity index values with and without sub-sampling. **Figures 5A, B**) show the changes in Hill’s diversity indices for different levels of sub-sampling. As seen in the figures, changes are consequential for values *α* <1 , which puts more weight on rare clonal populations. This finding confirms our expectations, i.e. lower sequencing depths fail to detect rare clonal clusters and result in lower estimations of diversity. On the other hand, no significant change is observed for common clusters, α>1 , which are detected even at low sequencing depths. We repeated the analysis using 2 clonal identification methods, the VJ & Junction and the alignment-free method (**Figures 5A, B** respectively). The same pattern is observed with both methods, with the alignment-free method resulting in a smaller change between the different sub-sampling ratios. **Figures 5C, D** shows the fold changes between Hill’s diversity index computed with a 10% sub-sampling and 100% sampling, repeated 100 times and averaged over repetitions. α=0 shows the highest variability, which is expected as this metric places equal weight on all clones regardless of their frequency. Singletons, whose detection strongly depends on sequencing depth, contribute to the observed high standard deviation associated with this metric. Similar to the previous figures, **Figures 5C, D** indicate that diversity metrics associated with α>1 , e.g. dominance, Shannon entropy, Simpson diversity index, etc., are only weakly affected by the sequencing depth.

The figures also indicate fold changes for the Chao estimators of richness and Shannon entropy (its exponential is shown in **Figures 5C, D** for consistency with the Hill’s framework, see Method Section 4.4.1). As these indicators aim to correct for sample size variability, we expect them to be less sensitive to changes in sequencing depth than their uncorrected equivalent (richness and Shannon entropy, respectively). However, this is not what we observe. For instance, the Chao estimator for richness shows more sensitivity to sequencing depth (fold change of 1.4) than richness itself (fold change 1.2), while the Chao estimator for Shannon entropy only yields moderate improvements of the sensitivity to sequencing depth. As the same pattern was observed on the other two datasets, we conclude that the Chao estimators for diversity indices results in minor improvements at best, and in some cases, might even reduce the accuracy of diversity estimation. As we discussed in the previous section, this is likely a consequence of the unreliable estimation of the number of singletons, which heavily affects the Chao estimators.

## 3 Discussion

B cells play a crucial role in the adaptive immune system, and their characterization can provide important clues about the immune status of an individual as well as about past and current infections or immune conditions. The advent of efficient experimental approaches for the high-throughput sequencing of BCR repertoires has generated unprecedented opportunities to unravel the dynamical changes that accompany complex B cell responses. However, with these new experimental opportunities have come significant challenges associated with the development of robust analytical approaches to characterize these data which can accurately shed light onto the underlying biological phenomena. In this paper we have investigated how the choice of different clonal identification methods and diversity metrics can bias the estimation of sample diversity.

The first step in the analysis of B-cell repertoires is the grouping of BCR sequences into B-cell clones that are expected to descend from a common ancestor cell, and therefore, share high sequence similarity. In this study, we compared the performance and potential biases associated with different clone identification methods and highlighted the potential drawbacks of methods that rely on germline gene alignments. We found that these methods can become unreliable for short read lengths, which can make the calling of the V gene inaccurate (Section 2.3). More importantly, we showed that the choice of the method can greatly impact the inferred clonal structure, especially for low-frequency and singleton clones (Section 2.4). This in turns might bias the analysis of immune repertoires in specific biological contexts.

Our analysis suggests that the VJ & Junction method remains the most accurate to identify clonal groups and singletons, while the junction-only performed worst on the simulated data. However, the choice of the clonal identification method should be made taking into consideration the experimental design and constraints of each dataset. For instance, we observed that if the V gene assignment is ambiguous, the alignment-free method proposed by Lindenbaum et al. (34) was a better choice to alleviate experimental limitations that result in incorrect V/J assignments. That is because this alignment-free method does not rely on the V/J assignments but rather compares the sequence similarity of the whole VDJ sequence with the help of vectorized representations of the BCRs. Overall, our results suggest that alignment-free strategies are a promising approach for B-cell clone identification and deserve further investigation.

Another important aspect we explored in this article is the impact of sequencing depth on the quantification of diversity. As clonal compositions across individual repertoires are highly variable, the analysis of repertoires by means of diversity indices offers the unique advantage of extracting biological information without directly comparing sequences across repertoires. We performed subsampling experiments and characterized the variability of different diversity metrics with sequencing depth and clonal identification methods. We analyzed the change in these metrics when different clonal identification methods were used and found that, while the absolute values were different, the main patterns of variation were conserved. In particular, the analysis of individual samples through diversity indices such as dominance, Shannon entropy, and richness led to high sample rank similarity. Shannon entropy was the most robust index (maximizing the Spearman sample rank correlation across methods) in the datasets and clonal identification methods we analyzed, which might be due to its weighting rare and abundant species similarly. Nevertheless, as different diversity indices provide different information, the best practice remains to combine several indices to gain a global view of diversity. In that respect, Hill’s diversity profiles already encompass information about many different indices, and therefore, already provides a more global understanding of B-cell repertoires than any given index. Finally, the use of Chao statistical estimators did not significantly lower the variability of the diversity estimation, both in terms of sub-sampling and clonal identification methods. As these estimators rely heavily on singletons and doubletons estimation, a potential cause behind these inefficiencies could be the unreliable detection of singletons during the process of clonal identification.

Considering the widespread variability we observed across datasets, methods and metrics, we can expect that the characterization of B-cell repertoire diversity will also show great variability in other applications. For instance, repertoires derived from blood or tissues samples typically showcase a high B-cell diversity, which is mostly composed of non-expanded B-cell clones. However, we can expect that more targeted applications, such as for the study of the immune responses induced by a foreign antigen (29) or the development of antibody libraries using phage display from a few starting B-cells (49, 50), exhibit lower diversity, as these systems are likely to result in a few dominant and highly expanded clones. Nevertheless, in these cases, where a repertoire is composed only by a handful of clones, identifying subclones (51) and characterizing the intraclonal diversity (52) may provide additional insight to the analysis.

In summary, we presented a quantitative comparison of different diversity metrics for the analysis of B-cell repertoires. We characterized the variability of these metrics when different clonal identification methods were used and for different sequencing depths. One of the main limitations of our analysis is the lack of ground truth in experimental datasets. To partially address this limitation, we included a synthetic dataset for which the ground truth is known by construction, which has enabled us to test the accuracy the different methods. However, addressing this limitation in an experimental context is much more difficult, and cannot be addressed in a fully satisfying manner yet. Rather, we leveraged *negation sequences* to estimate the specificity of the clone identification methods, i.e. random sequences extracted from different experimental studies, that are very unlikely to be clonally related to sequences in the considered study. However, negation only helps to set up the threshold between singletons and non-singletons. Quantifying the accuracy of the identified clones still remains a subjective endeavor. Nevertheless, we presented an overview of the different methods’ performances by evaluating the agreement between them (AMI). In particular, we investigated whether different methods agreed in the identification of singletons and found that the agreement was between 80% and 90% for the three datasets (SI section 1).

In future work, we aim to investigate whether additional improvements to the alignment-free method (34) can further boost its accuracy. For instance, the current alignment- free approach uses BCR vectorized representations based on k -mer frequency vectors, as posited by the *tf-idf* metric. This representation does not exploit potential semantic similarities between k -mers and assumes that the counts of different k -mers provide independent evidence of similarity. An important limitation of this approach is that the order of the k -mers in the sequence is not taken into consideration. Furthermore, the frequency vector is dataset-dependent, as the *tf-idf* metric computes frequencies across a corpus. Changing the dataset, i.e. the corpus, might result in changes in k -mers frequencies, and therefore in different clonal groupings. This limits the applicability of this metric across different repertoires. An alternative and attractive possibility to obtain vectorized representations of BCRs might be to leverage recent neural network and deep learning models for protein tasks, such as Immune2vec (53), ESM (54), TAPE (55), ProGen (56) or ProtBERT (57). The latent space of these pre-trained models can be used to readily extract a vector representing each BCR sequence. Given the more modest performance of the alignment-free method on the simulated dataset (AMI =0.84 ) compared to the VJ & Junction method (AMI =0.90 ), this could be a powerful tool to further improve the accuracy and scalability of current alignment-free clonal identification techniques.

In conclusion, the study of immune repertoires, particularly B cell repertoires, is critical for understanding the pathogenesis of various diseases and for the development of new diagnostic and therapeutic strategies. Our contribution in defining clonal diversity and diversity indices is an important step towards a better understanding of the immune system and its role in health and disease.

## 4 Methods

### 4.1 B cell repertoire preprocessing

Here, we detail here the preprocessing steps that we performed on all 3 datasets included in our study.

1. Data were downloaded from their original study: GC Data (36) hepatitis B vaccination data (8) and simulated repertoire data (42). Additionally, a set of the negation sequences was generated by randomly sampling sequences from multiple unrelated individuals.
2. For each sequence, the V and J genes were located and annotated based on the alignment to the germline genes downloaded from the IMGT reference directory sets (58), using IgBlast (46).
3. For the V and J gene assignments, we kept only the germline gene with the highest confidence from IgBlast. In the rare case where a sequence had multiple V and J genes identified with same confidence, we chose the first in alphabetical order.
4. Sequences were only retained if they were classified as *productive* by IgBlast.
5. Sequences with the same junction sequences were grouped together and represented by a single sequence randomly selected among them. Sequences within this group were considered to be clonally related because it is very unlikely that two sequences from the different clonal groups have exactly the same junction sequence (SHMs can occur in any part of the sequence and are not limited to the junction region). This assumption greatly reduces the computational workload.

### 4.2 Metrics

#### 4.2.1 Levenshtein distance

The Levenshtein distance (59) is defined as the minimum number of edits required to transform one sequence into another and is a common metric to quantify sequence similarity. To reduce the bias caused by length differences, we used the normalized Levenshtein distance (40) that incorporates the length of both sequences in the following manner:


(2) $${Lev}_{\text{norm}}\left(s_{1},s_{2}\right)=\frac{2\cdot Lev\left(s_{1},s_{2}\right)}{\left|s_{1}\left|+\right|s_{2}\right|+Lev\left(s_{1},s_{2}\right)},$$

where $\left|s_{1}\right|$ and $\left|s_{2}\right|$ are the lengths of strings $s_{1}$ and $s_{2}$ , and $Lev\left(s_{1},s_{2}\right)$ is the Levenshtein distance between these two strings.

#### 4.2.2 Cosine similarity

The cosine similarity is a measure of similarity between two vectors. Namely, given vectors **A** and **B**, the cosine similarity is defined as:


(3) $$\text{Cosine}\left(A,B\right)=\frac{A\cdot B}{\left\|A\|\|B\right\|}=\frac{\sum_{i=1}^{n}A_{i}B_{i}}{\sqrt{\sum_{i=1}^{n}A_{i}^{2}}\sqrt{\sum_{i=1}^{n}B_{i}^{2}}}\;.$$

To apply this similarity measure to BCR sequences, we first need to *encode* them. In this paper, we use the term frequency-inverse document frequency (*tf-idf*) weighting scheme. In brief, we first compute a k -mer representation of each BCR (substrings of length k )). Then, for each k -mer, the frequency term tf(k) is reweighted with the inverse document frequency, which is defined as $idf\left(k\right)=\log\;\left(\frac{\left|S\right|}{\left|k\in s,\;s\in S\right|}\right)$ , where |S| is the total number of sequences and the denominator is the total occurence of a specific k -mer k across all the S sequences. The final *tf-idf* representation is then computed as *tf−idf* (*k*) = *tf* (*k*) · *idf* (*k*). The logic behind this is to emphasize rare and hopefully meaningful terms while reducing the influence of common and uninformative terms.

#### 4.2.3 Adjusted mutual information

The mutual information (MI) of two random variables is a measure of the mutual dependence between these two variables. More specifically, it quantifies the “amount of information” obtained about one random variable by observing the other random variable. The mutual information of two jointly discrete random variables X and Y is calculated as:


(4) $$\text{MI}\left(X,Y\right)=\sum_{y\in Y}\sum_{x\in X}P_{\left(X,Y\right)}\left(x,y\right)\log\;\frac{P_{\left(X,Y\right)}\left(x,y\right)}{P_{X}\left(x\right)P_{Y}\left(y\right)},$$

where $P_{\left(X,Y\right)}$ is the joint probability mass function of X and Y , and $P_{X}$ and $P_{Y}$ are the marginal probability mass functions of X and Y, respectively (44).

MI can also be used to compare clusters, for instance, by measuring the information shared by the two clustering partitions. In practice, this is done by counting the number of sequences that are shared by each pair of clusters, $A_{i}$ and $B_{j}$ , where $A_{i}$ comes from the first clustering partition A and $B_{j}$ from the second ℬ :


(5) $$\text{MI}\left(A,ℬ\right)=\sum_{i\in A}\sum_{j\in ℬ}P_{\left(A,ℬ\right)}\left(i,j\right)\log\;\frac{P_{\left(A,ℬ\right)}\left(i,j\right)}{P_{A}\left(i\right)P_{ℬ}\left(j\right)},$$

The adjusted mutual information (AMI) is a modified version of the MI to compare two random clusters.

One limitation of the MI to compare partitions is that the baseline value of MI becomes larger when the number of clusters in both partitions increases. To address this limitation, the adjusted mutual information (AMI) can be used instead (44). Defining E{MI(U,V)} as the expected mutual information between two random clusters, the AMI is computed as:


(6) $$\text{AMI}\left(A,ℬ\right)=\frac{MI\left(A,ℬ\right)-E\left\{MI\left(A,ℬ\right)\right\}}{max\;\{H\left(A\right),H\left(ℬ\right)\}-E\left\{MI\left(A,ℬ\right)\right\}},$$

where H(A) and H(ℬ) are the entropies associated with the partitioning A and ℬ , respectively. With this transformation, the AMI takes a value of 1 when the two partitions are identical and 0 when the MI between the two partitions equals the value expected due to chance alone. We used the python implementation from sklearn to compute the AMI.

### 4.3 Identifying clones

We implemented three clonal identification methods in this article.

- **Baseline:** B cells were assigned to the same clone if and only if their receptors shared *exactly* the same CDR3 sequence.
- **VJ & Junction:** We first grouped B cells together if they had the same V and J gene. For each obtained group, we then computed the pairwise normalized Levenshtein distance between each junction in that group, and applied the Hierarchical Agglomerative Clustering (HAC) algorithm (39, 43) to cluster the BCRs into different clonal groups. We used the complete-linkage clustering criterion, which begins by clustering each sequence into its own cluster, and then sequentially combines smaller clusters into larger ones until all elements are in the same cluster. The complete scheme uses the maximum distances between all observations of the two sets to decide which clusters to merge next. This method results in a dendrogram that shows the sequence of cluster fusion and the distance at which each fusion took place. By setting an appropriate threshold, we can define individual clusters as all the clusters that have not been fused up to that distance. In this study, we chose as threshold the distance to the nearest distribution of negation sequences with a tolerance of 1%. In brief, the threshold is chosen such that it allows a fraction of false-positive sequences that is roughly equal to a tolerance *δ* to be below the chosen threshold. This heuristic aims for high specificity, which is approximately 1−δ .
- **Alignment-free:** All B cells sequences were first truncated from their $3^{\prime }$ end to a fixed number of nucleotides ( L ), and then encoded into a numerical vector using their k -mer representation reweighted with the term frequency-inverse document frequency (*tf-idf*) weighting scheme (see section 4.2.2). Following on from previous work (42), we set k=7 and L=130 as this combination was found to yield optimal performance in terms of clonal identification.

Next, we computed a distance matrix for all sequences in the repertoire using the cosine distance. Cosine distance has the advantage of being very fast to compute for sparse vectors, especially when compared to other alternatives, such as the Euclidean metric. Finally, the threshold definition and clustering into clonal groups were performed using HAC in the same way as in the VJ & Junction method.

### 4.4 Quantifying species diversity

#### 4.4.1 Diversity indices

Various indices, such as Shannon entropy (20), Simpson index (21), and species richness (38), are commonly used to quantify the *diversity* of an ecosystem. However, the choice of a universal index to objectively quantify and compare species diversity remains a topic of debate (60). Starting from the simple assumption that, when all species are equally common, diversity should be proportional to the number of species, Hill’s unified diversity framework (22) defines a general formula for the species diversity index that depends on an index α as follows:


(7) $${\;}^{\alpha }\text{D}={\left(\sum_{i=1}^{S}p_{i}^{\alpha }\right)}^{1/\left(1-\alpha \right)}$$

where $p_{i}$ is the relative abundance of species i , and $\sum^{}p_{i}=1$ . For a given number of species S>0 , one can prove that $1\leq^{\alpha }\text{D}\leq S$ .

The choice of α plays a role in the weighting of species of different frequencies. α<1 favors rare species, while α>1 favors common species. The most interesting aspect of Hill’s unified diversity index is that one can recover the most common diversity indices used in the literature for particular values of α , such as:

- **Species richness** ( α=0 ). The diversity of order zero is insensitive to species abundances and simply corresponds to the number of species: ${\;}^{0}\text{D}=S$

- **Dominance** ( α=∞ ). Diversity is sometimes represented as the proportion of its most abundant species $i_{\text{max}}$ , corresponding to the inverse of the infinite order diversity index.

(8) $$Dominance=\frac{1}{{\;}^{\infty }D}=p_{i_{\text{max}}\left(8\right)}$$

- **Shannon entropy** ( α=1 ). The Shannon entropy ( H ) weighs all species by the log  of their frequency. Although Eq. 7 is not defined when α=1 , its limit exists and converges to the exponential of the Shannon entropy (60).

(9) $${\;}^{1}\text{D}=\exp\;\left(\sum_{i=1}^{S}-p_{i}\log\;(p_{i})\right)=\exp\;(H)\;.$$

- **Simpson index** ( α=2 ). The Simpson index is defined as:

(10) $$\lambda =\sum_{i=1}^{S}p_{i}^{2}\;,$$

and represents the probability that two entities randomly selected from the dataset are of the same type (21). The Simpson index is directly related to the diversity of order two with $\lambda =\frac{1}{{\;}^{2}D}$ .

- **Evenness.** Rather than quantifying the diversity of species, the evenness (E) represents the homogeneity of abundances in a sample or a community (22). The evenness E(a,b) with orders a and b , a>b, is defined as

(11) $$\text{E}\left(a,b\right)=\frac{{\;}^{a}D}{{\;}^{b}D}$$

In practice, $\text{E}\left(1,0\right)=\frac{exp\;(H)}{S}$ is the most commonly used metric for quantifying evenness. Note that from this definition we always have 1S≤E(a,b)≤1 . In the case where the number of species is infinite, other values of (a,b) should be considered in order to obtain a non-zero evenness (22). Additionally, the E(1,0) evenness can be biased when the sample size is small, because it is sensitive to unobserved species. In this case, E(2,1) is preferred.

#### 4.4.2 Hill’s Diversity profile

Because Hill’s diversity profiles encompass the information contained in several diversity indices, its use is becoming increasingly common to obtain fingerprints of the immune repertoire (22, 23).

In this paper, we treated the *diversity profiles* as an N dimensional vector, where each element of the vector is the Hill’s diversity index ${\;}^{\alpha }\text{D}$ for a different value of α∈[0,∞] . Starting from a vector *A* with N elements in the range [−1,1] , we obtain the values of $\alpha =\left[\alpha_{1}\cdots \alpha_{N}\right]$ for our diversity profile with the transformation


(12) $$\alpha =\exp\;\left(\tan\;\left[A\frac{\pi }{2}\right]\right)$$

Then, the diversity profile ${\;}^{\alpha }\text{D}$ is computed as


(13) $${\;}^{\alpha }\text{D}=\left[{\;}^{\alpha_{1}}\text{D}\;\cdots {\;}^{\alpha_{N}}\text{D}\right]\;\;\;\text{where}\;\;{\;}^{\alpha_{k}}\text{D}={\left(\sum_{i=1}^{S}p_{i}^{\alpha_{k}}\right)}^{1/\left(1-\alpha_{k}\right)}\;.$$

We introduced that transformation to (i) be able to include both the Richness ( ${\;}^{0}\text{D}$ ) and Dominance ( $1{/}^{\infty }\text{D}$ ) in a finite vector, and (ii) to respect the symmetry between the weighting of species of different frequencies (where α<1 favors rare species, while α>1 favors common species).

#### 4.4.3 Estimating diversity with incomplete sample information

Complete knowledge about a system is often not available. Partial knowledge often results in the underestimation of a sample’s diversity, as some species might not have been observed. Specialized statistical tools have been developed to estimate the true richness $S_{\text{true}}$ , i.e. the true number of species, of a sample. One of the most common is the bias-corrected Chao1 species richness estimator (24, 25).


(14) $$S_{\text{Chao}}=S_{\text{obs }}+\frac{f_{1}\left(f_{1}-1\right)}{2\left(f_{2}+1\right)}\;,$$

where $S_{\text{obs}}$ is the total number of species detected, $f_{1}$ is the number of species detected exactly once, and $f_{2}$ the number of species detected exactly twice. The intuition behind this indicator is that if many species are detected only once, there is likely a large number of species that have not yet been detected. On the other hand, when all species have been detected at least twice, it is unlikely that new undetected species exist.

In addition to the Chao1 estimator for species richness, a similar approach can be used to estimate the Shannon entropy with incomplete sample information (48). Defining n as the number of observations, we can estimate the sample coverage as $C=1-\frac{f_{1}}{n}$ - , which represents a first order approximation based only on singletons, and adjust the relative species abundance with $\tilde{p_{i}}=p_{i}C$ . The Chao estimator for Shannon entropy can then be defined as:


(15) $$H_{\text{Chao}}=-\sum_{i=1}^{S_{\text{obs}}}\frac{\tilde{p_{i}}\text{log}\;\left(\tilde{p_{i}}\right)}{1-{\left(1-\tilde{p_{i}}\right)}^{n}}\cdot$$

Estimators can also be computed for higher orders of diversity, for instance, by using the general Horvitz-Thompson estimator (61) or other Chao estimators such as diversity rarefaction curves (26).

## Data availability statement

The original contributions presented in the study are included in the article/**Supplementary Material**. Further inquiries can be directed to the corresponding author.

## Author contributions

SL wrote the code and analyzed the data under the supervision of AP and MR. SL and AP wrote the manuscript. All authors contributed to the article and approved the submitted version.
