# Supplementary material for: Exploring the impact of clonal definition on B-cell diversity: implications for the analysis of immune repertoires
Source: Front Immunol. 2023 Apr 17;14:1123968. doi: 10.3389/fimmu.2023.1123968 (PMC10150052; doi:10.3389/fimmu.2023.1123968)
Supplement: Supplementary file 1 [file Presentation_1.pdf]

frontiers

Supplementary Material for: Exploring the Impact of Clonal Definition on B-Cell Diversity, Implications for the Analysis of Immune Repertoires

Aurelien Pelissier [1,2,*], Siyuan Luo [1,2,*], Maria Stratigopoulou [3], Jeroen EJ Guikema [3] and Maria Rodriguez Martinez [1,†]

[1]IBM Research Europe, 8803 Rüschlikon, Switzerland

[2]Department of Biosystems Science and Engineering, ETH Zurich, 4058 Basel, Switzerland

[3]Department of Pathology, Lymphoma and Myeloma Center Amsterdam (LYMMCARE), 1105 AZ Amsterdam, Netherlands

* Equal contribution

† Corresponding author: mrm@zurich.ibm.com

1 DISTANCE TO NEAREST DISTRIBUTION FOR EACH DATASET

The distance to nearest distribution is a key component in the identification of clones, as it is required to define the right threshold for the clustering. Unsurprisingly, the shape of the distribution, as well as the bi-modality of singletons and non-singletons looks different depending on the metric used (Figure S1).

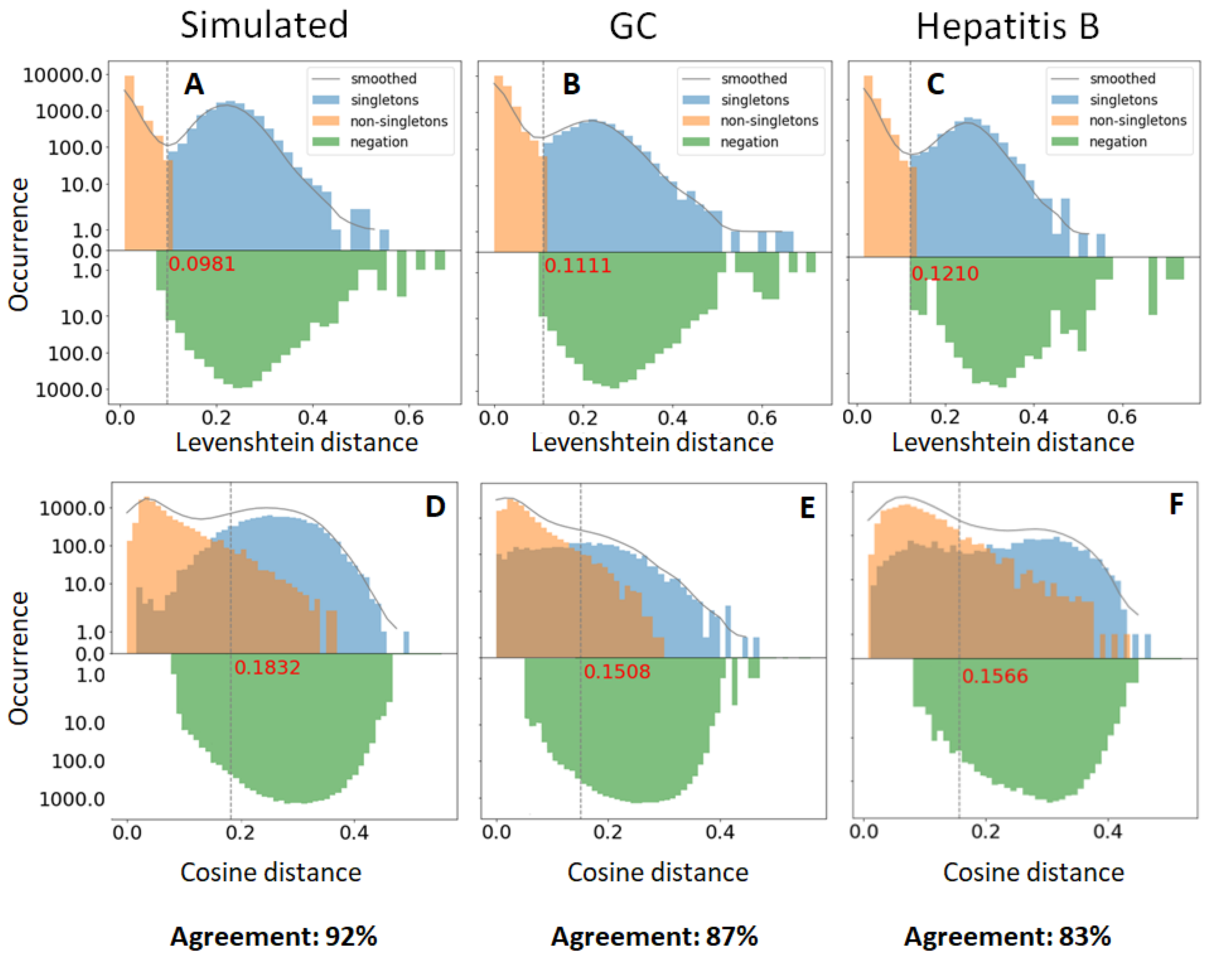

Figure S1: Distance to nearest distribution for each dataset in the context of both the alignment-based (Levenshtein distance) and alignment-free (Cosine distance) clonal identification method. In order to visualize the *agreement* between the two method (percentage of equal prediction), the singletons predicted by the first methods are used to label the distributions on the second method. The distance to nearest sequence in the negation dataset is also shown as a reference point. It was set with a tolerance of 1% for the alignment-free method

Interestingly, a subset of predicted singletons with a low Levenshtein distance in the alignment-based method have a high distance in the context of the alignment-free method. As a result, the singletons identified by both methods do not match exactly (percentage of equal prediction of 92%, 87% and 83% respectively across each dataset).

2 ROBUSTNESS ANALYSIS OF CLONAL IDENTIFICATION METHODS

One question of interest is how uncertain the clone assignment of a BCR sequence is in terms of the randomness in biological sampling. Since the comparison across different cell populations is non-trivial, we investigated the consistency of the clone identifier by comparing the clustering of the same subset of

sequences, but with/without the accompanying of the rest sequences as input. To do so, we sub-sampled each sample by randomly selecting half of its sequence, and then performed the clustering again with the subsampled sequences only (Figure S2A). After that, we compared the obtained clustering results to the original one (with the full sample) with the adjusted mutual information (AMI). In this case, the computed AMI represents the *robustness* of the clonal identification methods to biological sampling.

We performed this analysis for each sample and depicts the results Figure S2B. On average, the AMI of the junction-only, alignment-based and alignment-free method were $1, 0.92$, and 0.88 respectively. While our analysis indicates that all three methods have highly consistent clonal identification results, there is still significant differences between the three methods. As expected, the junction-only method always yields exactly the same results, since it do not rely on any clustering techniques. On the other hand, the alignment-free method is the least robust, as the BCR vectorized representation of sequences themselves changes with the subsampling (due to the learned *idf* reweighting).

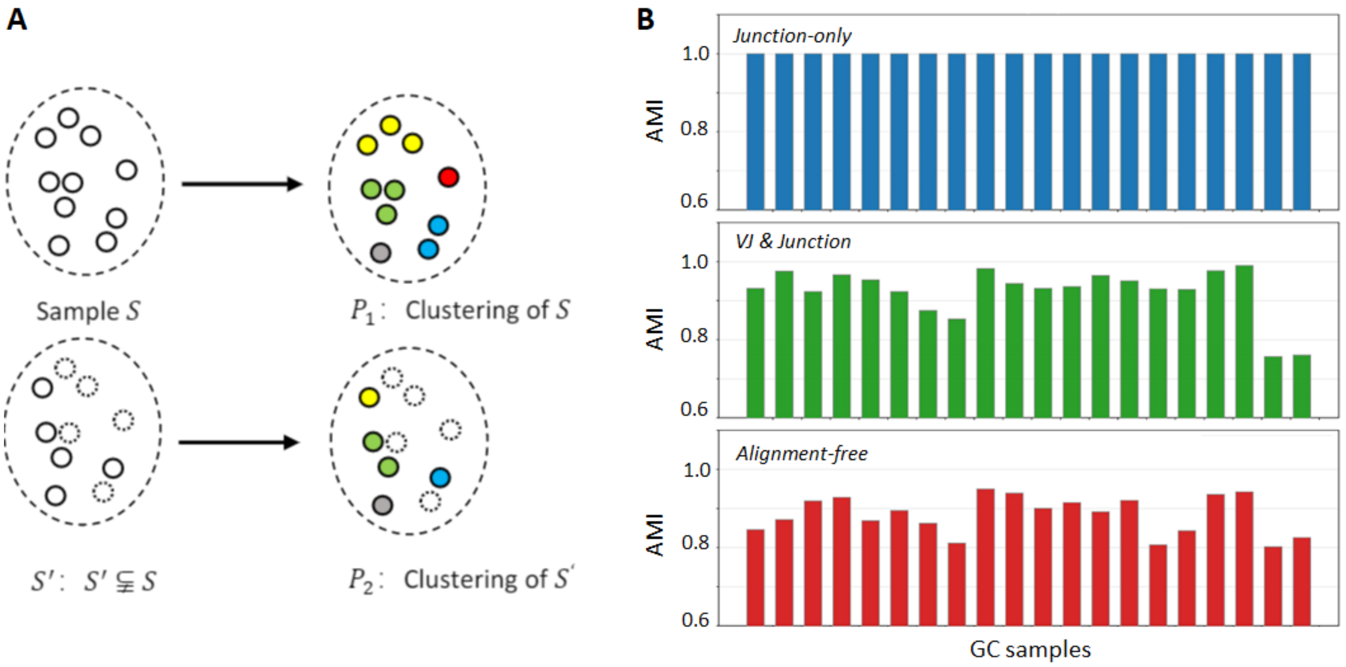

Figure S2: Robustness analysis of clonal identification methods. (A) A subsample S' is generated from sample S, and the obtained clonal families are compared across the two clustering. (B) Adjusted mutual information (AMI) between the original and subsampled clustering across each sample in the GC dataset, for the three clonal identification methods.

3 FALSE NEGATIVE SINGLETONS WITH THE ALIGNMENT-FREE METHOD

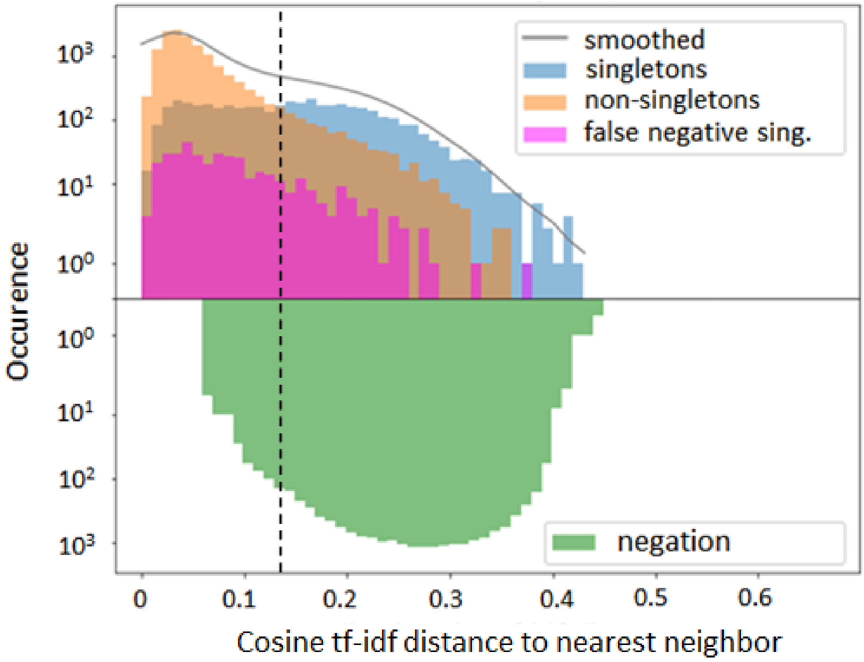

Figure S3: Distance to nearest distribution for B-cell sequences in the alignment free clonal identification method in the GC data. The sequences (singletons and non-singletons) are labeled with the alignment-based (VJ & Junction) method, where the false negative singletons were found from the ambiguous V or J gene misalignments (depicted in purple).

4 SPEARMAN CORRELATION OF DIVERSITY INDICES ACROSS METHODS

In Table S1, we provide the Spearman rank correlation for diversity indices in different biological context. First, we compare the diversity indices across the different clonal identification methods, which can be performed on the three datasets and that we extensively discuss in the main text. In the specific case of the simulated data, we can also test for the correlation between the diversity indices from different clonal identification methods and to ground truth diversity. Here we see that Shannon Entropy seems to perform the best in the context of different clocal identification. Finally, in the specific case of the germinal center dataset, we can test for the consistency of the diversity indices across replicates for each clonal identification method (we have two replicates per GC). The results Table S1 suggest that the Simpson index seems to be optimal in the context for the GC replicates (maximizing correlation averaged over the three replicates).

	Diversity index	JO *vs* VJJ	JO *vs* AF	VJJ *vs* AF	JO *vs* G_0	VJJ *vs* G_0	AF *vs* G_0	*Mean*
Simulated dataset	Richness	0.89	0.82	0.84	0.79	0.82	0.84	*0.83*
	Chao Richness	0.69	0.64	0.56	0.63	0.89	0.72	*0.69*
	Shannon Entropy	0.92	**0.88**	0.70	0.69	0.88	0.84	*0.82*
	Chao Shannon Entropy	**0.96**	0.80	0.83	0.84	**0.91**	**0.89**	*0.87*
	Simpson index	0.94	0.70	0.69	0.65	0.75	0.64	*0.73*
	Dominance	0.82	0.87	0.70	0.72	0.79	0.80	*0.78*
	Evenness	**0.96**	**0.88**	**0.84**	**0.85**	**0.91**	0.87	***0.89***
	Mean	*0.88*	*0.80*	*0.74*	*0.74*	*0.85*	*0.80*	

	Diversity index	JO *vs* VJJ	JO *vs* AF	VJJ *vs* AF	repl. JO	repl. VJJ	repl. AF	*Mean*
GC dataset	Richness	0.79	**0.97**	0.76	0.47	0.94	0.90	*0.81*
	Chao Richness	0.82	0.96	0.77	0.56	0.64	0.81	*0.76*
	Shannon Entropy	**0.99**	0.91	**0.89**	0.85	**0.96**	0.82	*0.90*
	Chao Shannon Entropy	**0.99**	0.90	**0.89**	0.85	**0.96**	0.84	*0.91*
	Simpson index	0.97	0.90	0.88	**0.95**	0.92	**0.96**	***0.93***
	Dominance	0.91	0.92	0.86	0.88	0.93	0.95	*0.91*
	Evenness	0.63	0.71	0.52	0.66	0.75	0.61	*0.65*
	Mean	*0.87*	*0.90*	*0.80*	*0.75*	*0.87*	*0.84*	

	Diversity index	JO *vs* VJJ	JO *vs* AF	VJJ *vs* AF	*Mean*
Hepatitis B dataset	Richness	0.98	0.98	0.94	*0.97*
	Chao Richness	0.87	0.96	0.80	*0.88*
	Shannon Entropy	**1.00**	**0.99**	**0.99**	***0.99***
	Chao Shannon Entropy	**1.00**	**0.99**	**0.99**	***0.99***
	Simpson index	**1.00**	**0.99**	**0.99**	***0.99***
	Dominance	**1.00**	0.98	0.97	*0.98*
	Evenness	0.99	**0.99**	0.96	*0.98*
	Mean	*0.98*	*0.98*	*0.95*	

Table S1. Spearman correlation of the diversity indices values across samples for each dataset. The correlation is computed between each pair of clonal identification method. *Junction-only* (JO), *VJ & Junction* (VJJ) and *Alignment-free* (AF). For the simulated dataset, comparisons are also performed on ground truth (G_0) clonal groups. For the GC dataset, we also compute the correlation between the GC replicates (designated as repl). Max values for each columns are highlighted in bold

5 VARIATION IN DIVERSITY ACROSS CLONAL IDENTIFICATION METHODS AND DATASET

We computed the diversity indices of each sample in each dataset and we show the results for Shannon entropy Figure S4A. Clearly, the variability of the Shannon diversity relative to the average value is not the same across sample. The simulated dataset show small variability in the Shannon entropy (mean/std ~ 80) while the Hepatitis B and GC dataset show higher variability (mean/std ~ 3 and ~ 1.5, respectively). This is reflected on Figure S4B, where the Hill's diversity profiles becomes near superposed for $\alpha < 1$. Thus, in

cases of low sample variability like these, ranking the samples Hill's diversities for these values of α might not be consistent.

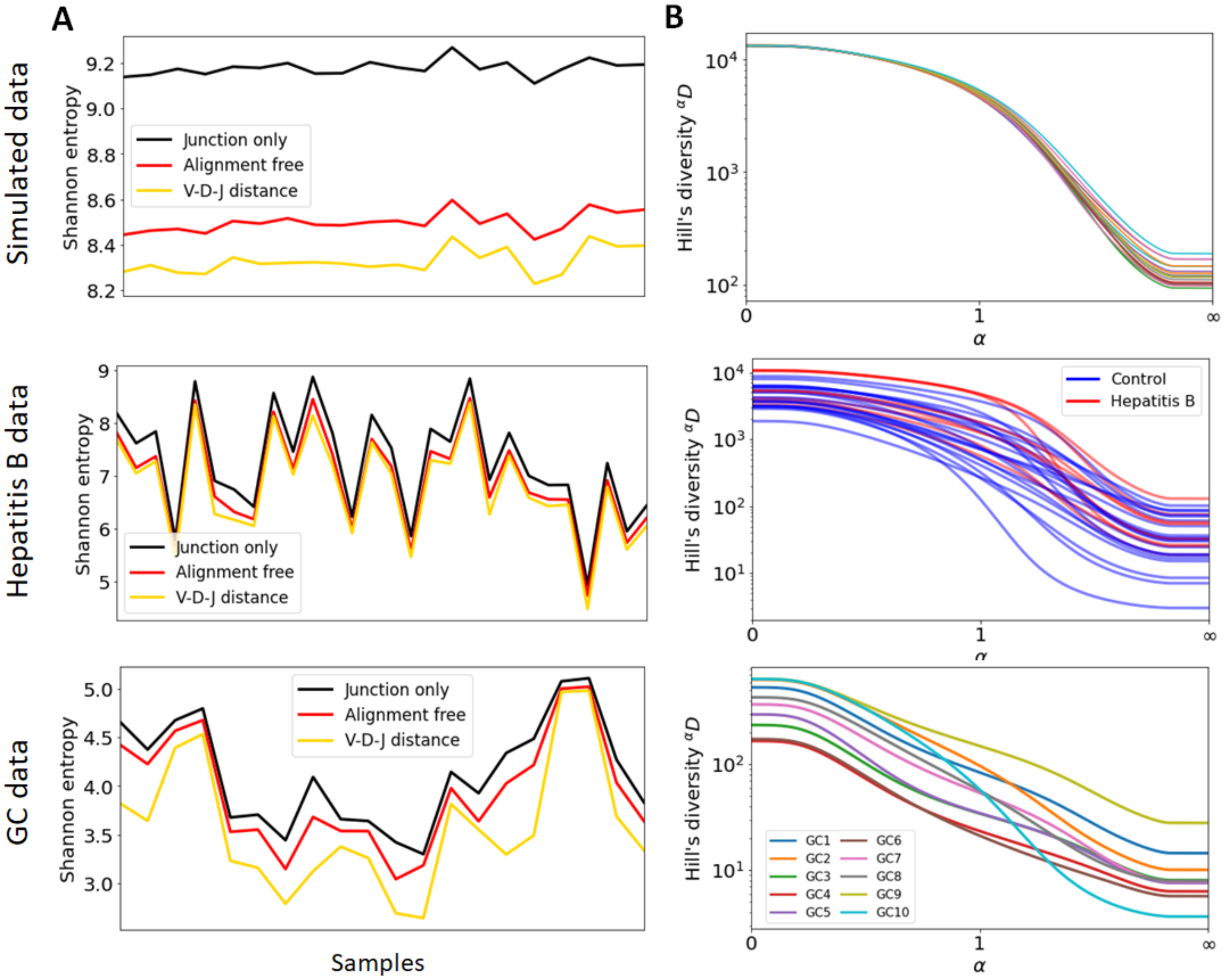

Figure S4: B-cell repertoire clonal diversity analysis. (A) Shannon entropy of each sample in each dataset for the three clonal identification methods. (B) Hill's diversity profile of each sample in each dataset, with clones inferred from the alignment-free method. Note that the x axis was transformed by a exponential tangent function for visual clarity.

On Figure S4A, we see that, not only different clonal identification method may yield to different values of diversity indices, the magnitude of the difference relatively to the variability in the index varies considerably across sample. To quantify the systematic inconsistencies in different diversity metrics between the JO, VJJ and AF clonal identification method, we computed the mean absolute difference of the metric between the two methods and normalized by the standard deviation of that metric on that dataset. A score below 0.1 indicate that the metric is overall consistent across clonal identification methods as the variation across method is much lower than the variation of the score itself. On the other hand, a score of the order of one or greater means that the effect of the clonal identification method on the diversity characterization cannot be neglected.

We computed this score for each method pairs for each metric in each dataset (Figure S5). Interestingly, metrics less sensitive to singletons such as dominance or Simpson index were less affected by the clonal identification method than those sensitive to rare clones (richness). Because the Hepatitis B dataset showcase huge variability in its clonal composition, it is less affected by changes in the clonal identification method (inconsistency < 1.1). Still, it is challenging to know a-priori which dataset will be more or less sensitive to clonal identifications, therefore we caution against comparing diversity indices across B cell clone repertoires in which different diversity indices were used.

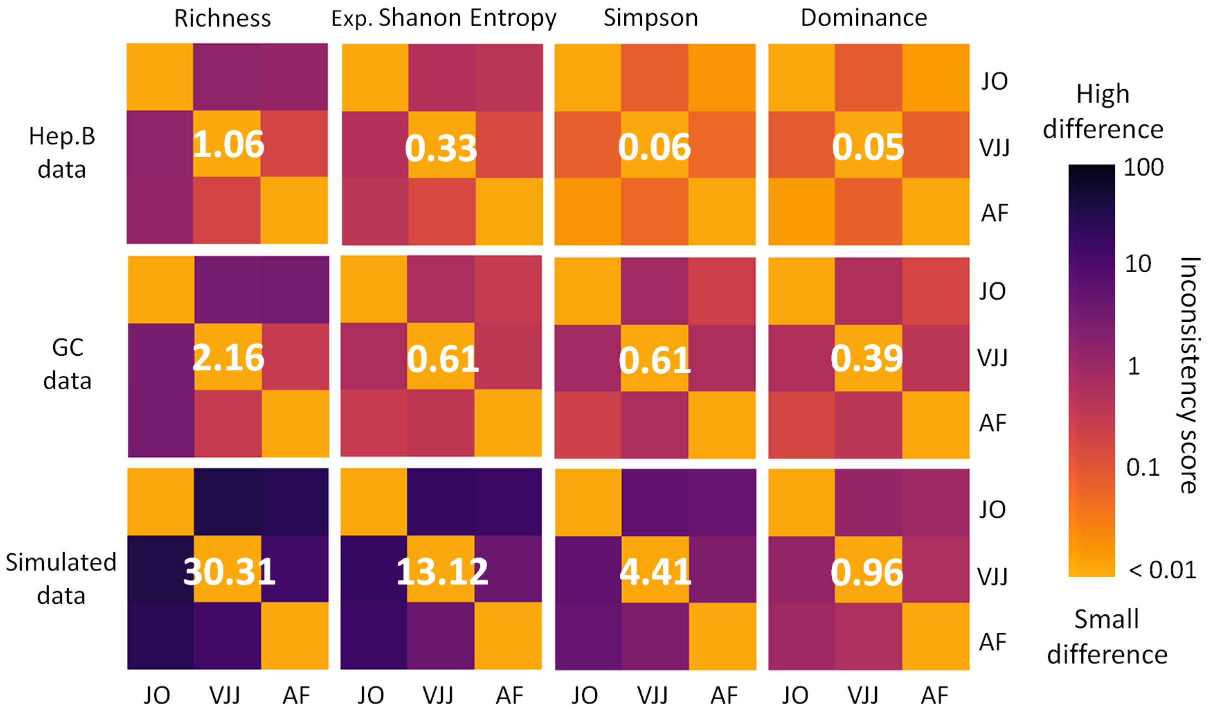

Figure S5: Pairwise matrix of the *inconsistency scores* between the JO, VJJ and AF clonal identification methods for each metric in each dataset. The score is computed as the mean absolute difference of the metric between the two methods and normalized by the standard deviation of that metric on that dataset. The numbers in white are the average of the score over non-diagonal elements.

6 CLONAL IDENTIFICATION PERFORMANCE WITH SUBSAMPLING

In section 1.2 of the main text, we quantified the accuracy of clonal identification methods on the simulated dataset with the AMI between the inferred clones and the ground truth labels. As the clusters of VJJ and AF methods rely on hierarchical clustering, the obtained clusters depend on the sequences present in the repertoire. Thus, the sequencing depth may affect the clustering performance. On Figure S6A, we show how the clustering accuracy varies with different subsampling of the data. We observe that, for subsampling greater than 10% (which corresponds to a sample size of $\sim$4k samples in the case of simulated data), the AMI stays consistent. Note that, while the AMI is lower for the smallest subsampling fractions, the same pattern for the JO method, which is independent on other sequences when clustering, was observed. Therefore, one could argue that the observed decrease in AMI for low subsampling fractions is an artifact of having low number of sequences rather than an actual decrease in performances. To further support this argument, we observe the same trend with the adjusted rand index (ARI) (Figure S6B).

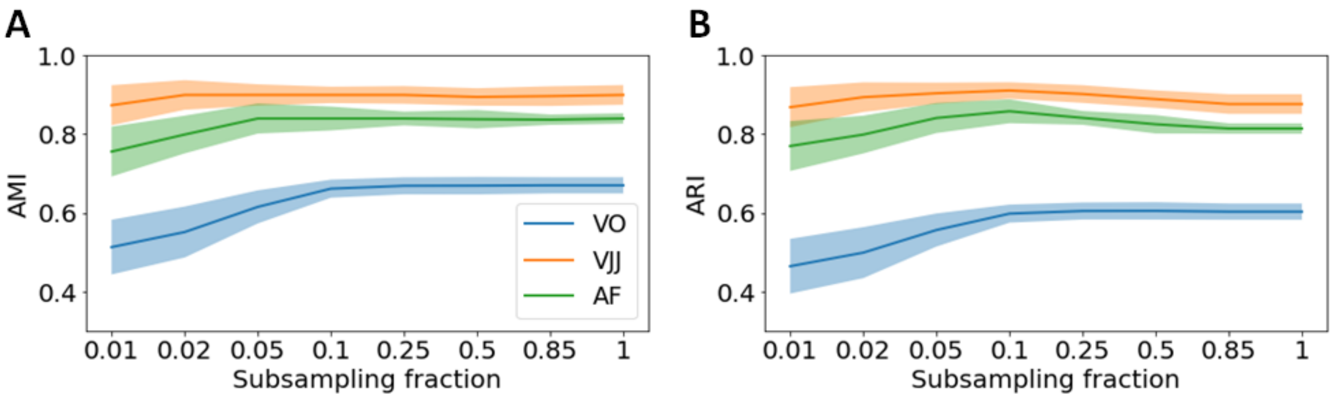

Figure S6: Clone identification performance on the simulated dataset for different subsampling, quantified in terms of (A) adjusted mutual information (AMI) and (B) adjusted rand score (ARI) to the ground truth labels. The subsampling was repeated 20 times and the shaded area indicates one standard deviation.
